# Editorial: Advances in aortic imaging

**DOI:** 10.3389/fcvm.2023.1137949

**Published:** 2023-02-01

**Authors:** Joseph R. Leach, Chengcheng Zhu, Nicolas Burris, Michael D. Hope

**Affiliations:** ^1^Department of Radiology and Biomedical Imaging, University of California, San Francisco, San Francisco, CA, United States; ^2^Department of Radiology, University of Washington, Seattle, WA, United States; ^3^Department of Radiology, University of Michigan, Ann Arbor, MI, United States; ^4^California Advanced Imaging Medical Associates, San Francisco, CA, United States

**Keywords:** aorta, imaging, radiology, aortic (rupture) dissection, aneurysm (abdominal aorta thoracic aorta)

The aorta is affected by a wide array of pathologies reflecting its size, central position in the vascular tree, and uniquely wide range of hemodynamic forces along its length (1–3). Accordingly, a breadth of powerful imaging tools are increasingly used in the evaluation of acute and chronic aortic diseases and to investigate the pathophysiology of disease initiation, progression, risk stratification, and aid in the development of new therapeutics (4–8). In this Research Topic “*Advances in Aortic Imaging*,” we have aimed to highlight for the cardiovascular community a number of potentially impactful new developments in aortic imaging spanning applications in clinical research, to rapidly-evolving translational imaging techniques, to new technical advances in image analysis.

Aneurysmal dilation of the aorta has long been a focal point for clinicians and researchers concerned with cardiovascular disease. In fact, “aortic aneurysm” encompasses several distinct pathologies with overlap of some risk factors but not others, disparities in morphology and anatomic extent, and differences in treatment. Abdominal aortic aneurysms (AAA), for instance, are the most common manifestation of aortic aneurysm, frequently associated with atherosclerosis and related to inflammation and mural matrix degradation (1, 9, 10). Thoracic aortic aneurysms (TAA) are substantially more diverse and are associated with intrinsic aortic wall fragility that may result from advanced age, hypertension, genetic abnormalities (e.g., Marfan syndrome), inflammation (e.g., atherosclerosis or aortitis), and flow-mediated aortic wall remodeling as with bicuspid aortic valve (BAV) (11, 12). Given the relative severity of TAA complications, such as dissection and rupture, and complexity of repair, an impressive body of imaging research, some included in this Research Topic, has targeted improved understanding of this disease and its complications.

In the study by Sotelo et al., 4D Flow MRI velocity fields were interpolated over the thoracic aorta using finite element techniques to characterize altered hemodynamics in patients with BAV, the most common congenital heart defect in which ~50% of patients develop ascending TAA (13, 14). Several flow metrics demonstrated associations with BAV compared to healthy controls, including flow eccentricity and velocity angle, regurgitation fraction, and the circumferential component of wall shear stress, while forward flow velocity and other metrics showed the best relationship with local aortic diameter. The comprehensive analysis methodology presented in this work is well-suited to larger prospective investigations on the hemodynamic underpinnings of ascending TAA development in BAV.

Aortic dissection (AD) (Figure 1) is among the most serious complications of TAA, and while modern CT angiography provides nearly perfect detection (15), reliable availability of contrast-enhanced imaging is not universal in some settings. Yi et al. studied advanced methods of AD detection on non-contrast CT, utilizing a combination of deep learning and automated aortic morphological analysis. Addition of morphologic metrics to a deep learning-based detection of AD improved prediction area under curve (AUC) from 0.814 to 0.969 in an external validation cohort of *N* = 117 (*p* < 0.05). The overall accuracy of their “deep integrated” model was comparable to that of a panel of three radiologists, though the specificity of their model reached only 0.55 suggesting that this technique may be most useful for identifying patients who may benefit from further evaluation. Such computer-aided assessments may help stratify patients in a high throughput screening paradigm, but deep learning—and perhaps even radiologists—may not be ready for AD detection on non-contrast imaging, although this work may serve as a basis for future tuning of deep learning models.

**Figure 1:**
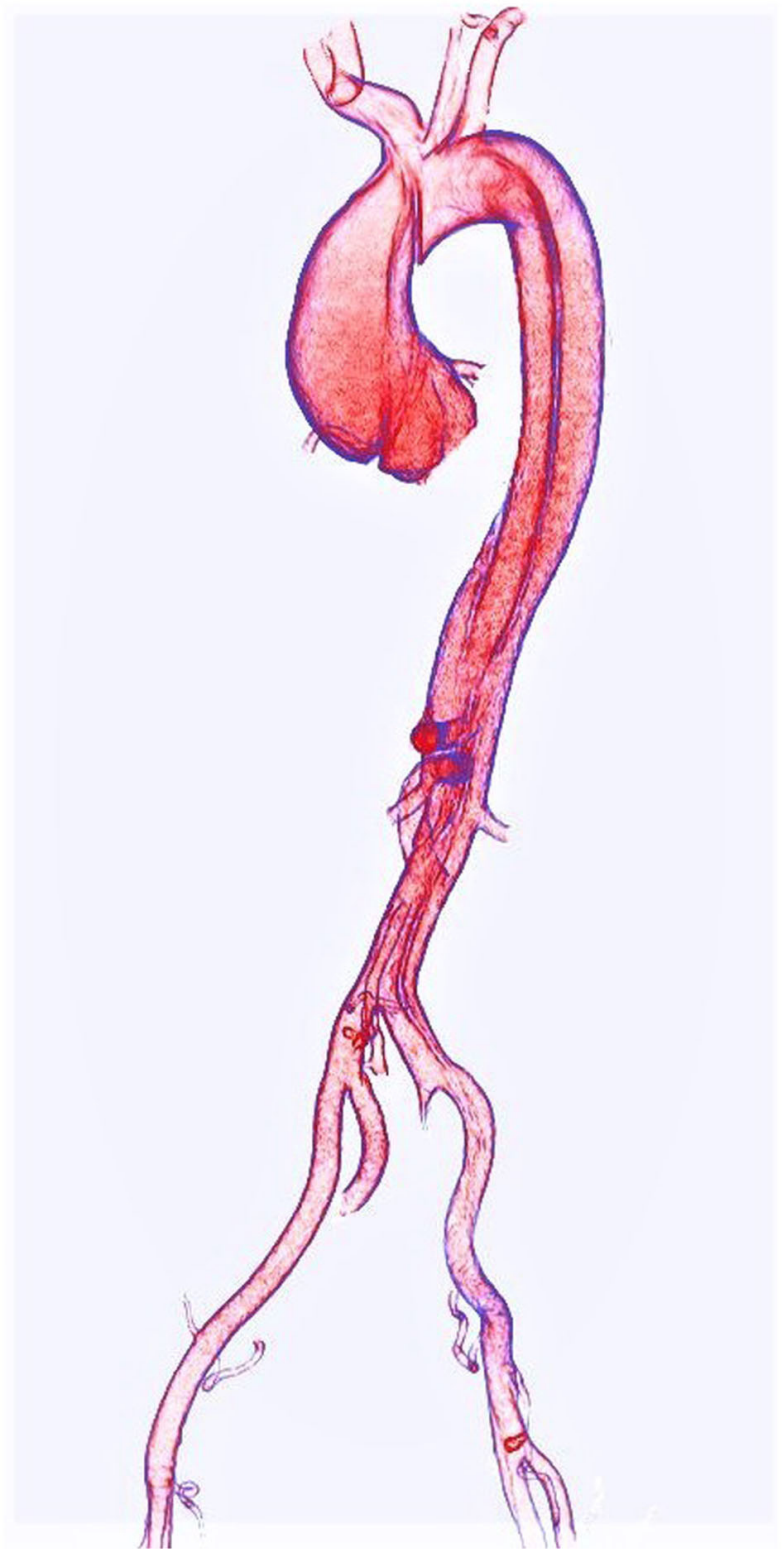
Volume rendering of an acute Type A aortic dissection as seen on CTA.

Prophylaxis against or repair of dissection of the dilated ascending aorta consists of surgical replacement of the diseased segment with a synthetic aortic graft (2). The limited distensibility of such grafts is hypothesized to cause potentially deleterious changes to ventriculoarterial coupling (16). In Houben et al.'s contribution to this collection, the effects of proximal aortic graft repair on left ventricular remodeling were explored using semiautomated segmentation of left ventricular myocardium on CTA. The study found that left ventricular myocardial index (LVMI) of surgically repaired aneurysm patients increased significantly within 9 months following repair, while the LVMI of medically managed aneurysm patients was unchanged after a follow-up period of ~14 months. While this study does not primarily focus on the imaging technique itself, it highlights the important role that advanced aortic imaging plays in multi-disciplinary research that spans surgical and engineering topics.

Unlike ascending aortic dissection (TAAD), uncomplicated dissection of the descending thoracic aorta (TBAD) is typically managed medically (17). Progressive aneurysmal dilation of the dissected aorta is a major precipitant of adverse outcomes, and an indication for life-long imaging surveillance (18). In their work, Chu et al. used 4D Flow MRI to characterize true- and false-lumen hemodynamics in 51 patients with isolated TBAD or residual type-B dissection after TAAD repair. *N* = 12 patients developed adverse outcomes, and these patients had a larger baseline aortic diameter and significant differences in multiple flow metrics, including false-lumen stasis, true-lumen kinetic energy and true-lumen peak velocity. In 42 of the patients with at least 180 days of imaging follow-up, those with rapid aortic growth (≥3 mm/yr) had a higher false lumen to true lumen kinetic energy ratio. While further prospective studies are needed to verify the predictive value of such metrics, Chu et al.'s work lends further support for the important role that advanced aortic flow imaging can play in risk stratifying patients with aortic dissection.

For complicated TBAD, thoracic endovascular aortic repair (TEVAR) is the most common treatment (17), although the effects on aortic blood flow induced by deployment of an endovascular graft within an already complicated hemodynamic environment remain poorly studied (18). Cosset et al.'s study in seven TBAD patients used pre- and post-repair 4D Flow MRI to elucidate these hemodynamic changes. Quantitative analysis of the 4D Flow field showed that TEVAR significantly increased forward flow in the true lumen and decreased both forward and backward flow in the false lumen. Reverse flow in the true lumen increased after TEVAR, possibly due to the relatively lower stent-graft distensibility compared to native aorta. The authors also noted that post-processing of the flow velocity data to visualize flow helicity allowed for improved identification of secondary entry tears both pre- and post-TEVAR. What such TEVAR-induced hemodynamic changes mean for long term durability of repair and remodeling of the false lumen remains unclear, but this work provides important early evidence of the hemodynamic effects of endovascular techniques that are increasingly used to treat TBAD patients.

These contributions highlight only a small fraction of the recent advances in aortic imaging and are complimented by additional works targeting both practical and fundamental gains in our understanding and treatment of aortic disease, from Peng et al.'s work to streamline aortic geometry assessment, to Maas et al.'s study on the natural history and significance of focal intimal flaps and Zhong et al.'s study on the progressive maturation of fetal aortic elastomechanics. We think “*Advances in Aortic Imaging*” gives an exciting and broad glimpse into the state of the art in the field, and the studies in this collection will serve as springboards to further investigation and new applications.

## Author contributions

All authors listed have made a substantial, direct, and intellectual contribution to the work and approved it for publication.
